# First person – Vanessa Gomez

**DOI:** 10.1242/dmm.052550

**Published:** 2025-07-28

## Abstract

First Person is a series of interviews with the first authors of a selection of papers published in Disease Models & Mechanisms, helping researchers promote themselves alongside their papers. Vanessa Gomez is first author on ‘ Distinguishing *PEX* gene variant severity for mild, severe, and atypical peroxisome biogenesis disorders’, published in DMM. Vanessa is a Research Assistant in the lab of Michael F. Wangler at Baylor College of Medicine, Houston, TX, investigating rare human disease phenotypes to advance our understanding of biological principles that govern health and disease.


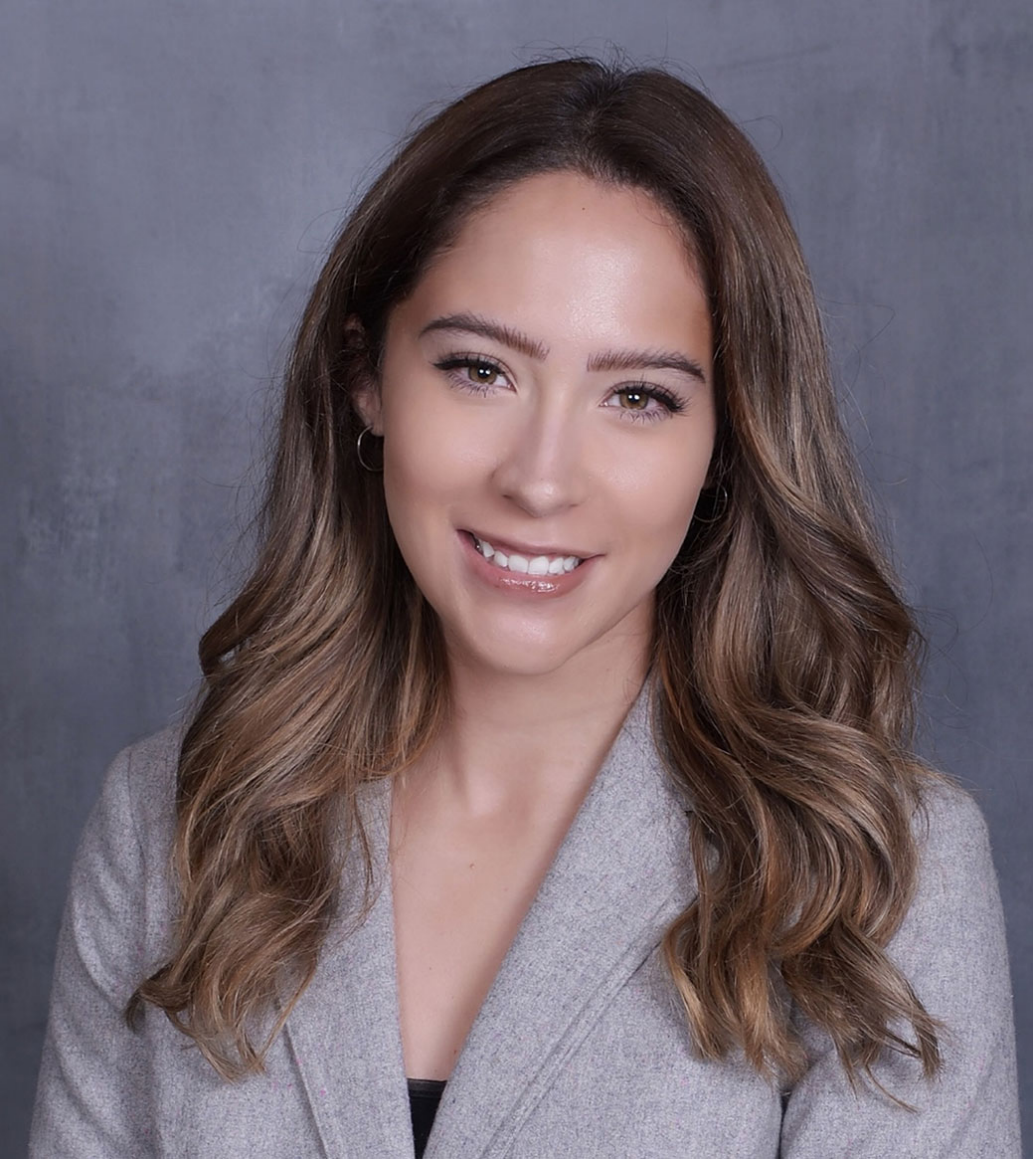


**Vanessa Gomez**

**Who or what inspired you to become a scientist?**

From a young age, I was drawn to science by a natural curiosity to understand how things work, especially the mechanisms behind human health and disease. I've always been motivated by a desire to ask meaningful questions and seek answers that can improve the lives of others. Along the way, I've been fortunate to be guided and inspired by professors, mentors and investigators who supported my passion for human health and shaped my path towards a career that bridges clinical phenotypes and disease mechanisms with scientific discovery.

**What is the main question or challenge in disease biology you are addressing in this paper? How did you go about investigating your question or challenge?**

Peroxisomal biogenesis disorders (PBD) are severe, multisystemic conditions caused by mutations in *PEX* genes, which are essential for forming and maintain peroxisomes. Despite having mutations in the same gene, patients can experience a wide range of disease severity, from lethal infantile presentations to mild ataxic phenotypes in adults. Previous research has shown that clinical severity often correlates with specific alleles, which suggests that some mutations retain partial function while other cause complete loss. While clinical data supports this idea, we lack a scalable *in vivo* system to directly assess how specific *PEX* mutations affect peroxisomal function. This gap in knowledge led us to investigate whether we can functionally characterize individual human *PEX* variants *in vivo* to understand why they cause variable disease phenotypes and establish an effective model of disease.

Our study highlights the value of using *Drosophila* as a model to functionally test human mutations and refine genotype−phenotype correlations.

To address this, we developed a *Drosophila*-based functional model that allows us to test the impact of individual human *PEX* variants *in vivo*. We utilized *KozakGAL4* cassettes to knock-out *Pex* genes of interest, while simultaneously inserting a *GAL4* driver to create *Pex* null fly alleles that could be ‘rescued’ with human gene expression. We generated UAS-cDNA lines for human *PEX2* and *PEX16* reference sequences as well as patient-derived variants and expressed them in fly null backgrounds to assess whether the human proteins can functionally replace the fly gene. We performed lifespan, bang sensitivity and climbing assays to assess rescue of viability, and disease progression. The phenotypes observed allowed us to construct a functional severity spectrum of human alleles. This model allowed us to confirm that some missense and nonsense variants behave as severe as null mutations, as well as identify alleles with partial rescue to support their classification as a mild mutation. Our study highlights the value of using *Drosophila* as a model to functionally test human mutations and refine genotype−phenotype correlations.

People with mutations in the same gene can have very different symptoms. Some patients get very sick and die within the first year of life, while other patients live into adulthood with mild symptoms.

**How would you explain the main findings of your paper to non-scientific family and friends?**

We studied a group of rare genetic diseases caused by changes in particular genes that help the detoxification center of our cells, called peroxisomes, to develop and do their job effectively. These types of disorder are called peroxisomal biogenesis disorders (PBD). What is unusual about this disease is that people with mutations in the same gene can have very different symptoms. Some patients get very sick and die within the first year of life, while other patients live into adulthood with mild symptoms.

To understand why this happens, we used fruit flies to study this human disease. We removed the fly version of the gene and added in different human versions of the gene, some from healthy people not affected by the disease, some with mild versions of the disease and some with a severe version of the disease. We then were able to observe how well the flies did with the different versions of the gene. We found that some of the human gene changes were just as harmful as having no gene at all, while other gene changes worked partially or almost normally. This helps explain why the disease looks so different from person to person and gives us a way to test how risky a gene change might be in the future.

**What are the potential implications of these results for disease biology and the possible impact on patients?**

Our study provides key insights into the functional consequences of specific *PEX2* and *PEX16* variants associated with PBD. By generating humanized *Drosophila* models, we were able to directly assess how individual human alleles impact peroxisome function, viability and neurobehavioral phenotypes *in vivo.* Our findings support the existence of an allelic severity spectrum for both *PEX2* and *PEX16* by using a *Drosophila* model*,* which allows for systematic testing of variants to refine the correlation between molecular defects and clinical outcomes. This will be especially useful for atypical phenotypes in patients that fall outside the traditional PBD classification, such as isolated ataxia or Heimler syndrome. This work advances both mechanistic understanding of peroxisomal disease and provides a translatable tool for precision medicine approaches in the future for rare genetic disorders.

**Figure:**
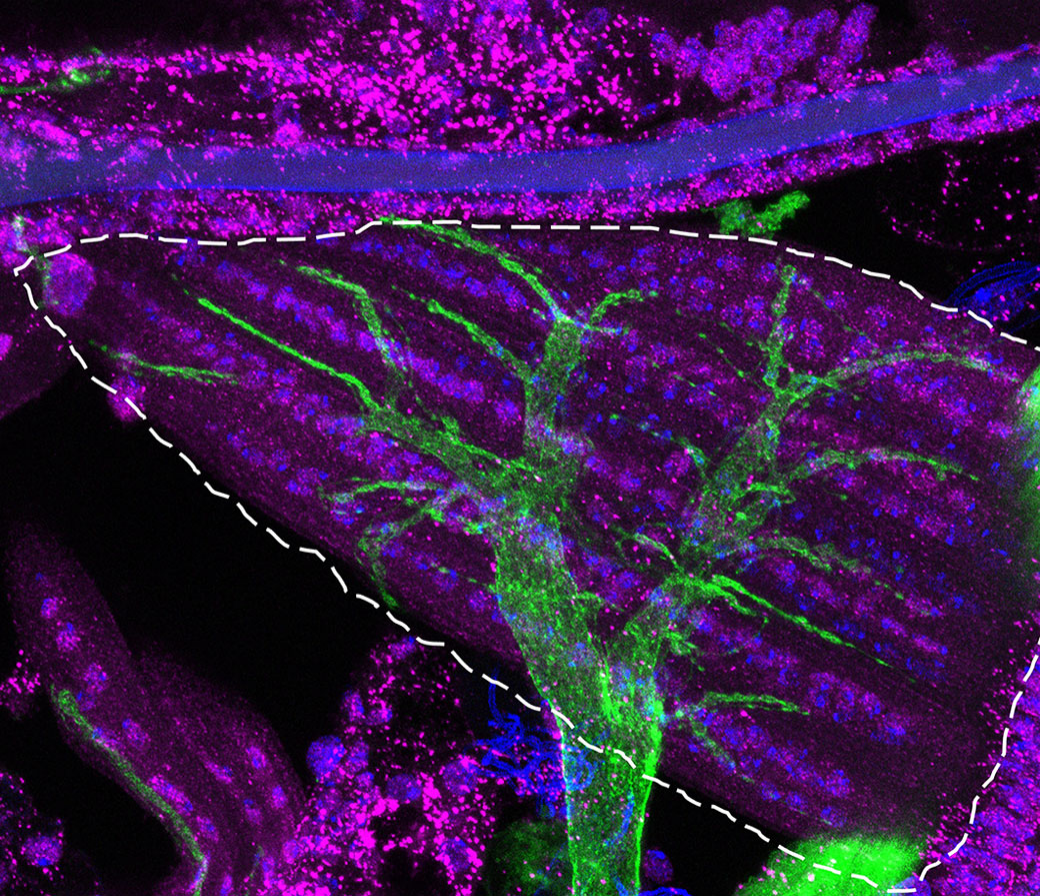
**Morphology of the direct flight muscle in *Pex2* mutant flies indicates a reduction in Pex3 staining and apparent thickening of the surrounding nerve fiber in 0-day-old adult flies.** This morphological finding indicates peroxisomal disease progression in the fly.

**Why did you choose DMM for your paper?**

DMM is a highly regarded, Open Access journal that focuses on using model organisms to study human disease, which aligns perfectly with the goals of our work. Our study uses *Drosophila* to functionally characterize human *PEX* gene variants and provide a translatable tool for rare genetic disorders. The journal's diverse readership is ideal for sharing our findings with fellow scientists and those interested in rare genetic disorders.

**Given your current role, what challenges do you face and what changes could improve the professional lives of other scientists in this role?**

The biggest challenge I've observed in my current role is the uncertainty surrounding research funding. Scientific progress depends on sustained efforts, but funding sources can often be unpredictable and make it difficult to invest in innovative ideas. Improving the stability of funding could go a long way in supporting early-career researchers and fostering an innovative research environment.

**What's next for you?**

I'm excited to take the next step in my journey toward a more patient-centered role and apply to graduate school. My goal is to build on my research background by moving into a clinical setting where I can more directly impact individual lives and bridge the gap between bench research and patient care.

**Tell us something interesting about yourself that wouldn't be on your CV**

Outside of the lab, I'm passionate about staying active, particularly running and practicing yoga. For me, exercise is as much about mental clarity as it is about physical strength. I find that staying active clears my mind and allows me to maintain a balanced lifestyle, which is crucial for staying sharp and showing up as my best self in both my personal life and scientific work.
